# The Role of Different Indexes of Adiposity and Body Composition for the Identification of Metabolic Syndrome in Women with Obesity

**DOI:** 10.3390/jcm10091975

**Published:** 2021-05-04

**Authors:** Giorgio Radetti, Antonio Fanolla, Graziano Grugni, Fiorenzo Lupi, Sofia Tamini, Sabrina Cicolini, Alessandro Sartorio

**Affiliations:** 1 Marienklinik, 39100 Bolzano, Italy; 2 Observatory for Health Provincial Government, 39100 Bolzano, Italy; Antonio.Fanolla@provinz.bz.it; 3 Division of Auxology, Istituto Auxologico Italiano, IRCCS, 28824 Piancavallo, Italy; g.grugni@auxologico.it (G.G.); sartorio@auxologico.it (A.S.); 4 Experimental Laboratory for Auxo-Endocrinological Research, Istituto Auxologico Italiano, IRCCS, 28824 Piancavallo, Italy; s.tamini@auxologico.it (S.T.); s.cicolini@auxologico.it (S.C.); 5 Department of Pediatrics, Regional Hospital of Bolzano, 39100 Bolzano, Italy; fiorenzolupi@gmail.com; 6 Division of Metabolic Diseases, Istituto Auxologico Italiano, IRCCS, 28824 Piancavallo, Italy

**Keywords:** adiposity indexes, metabolic syndrome, obesity

## Abstract

The aim of this study was to compare the accuracy of different indexes of adiposity and/or body composition in identifying the metabolic syndrome (MetS) in a group of 1528 Caucasian women with obesity: (age ± standard deviation (SD): 50.8 ± 14 years (range 18–83); body mass index (BMI) 43.3 ± 5.9 kg/m^2^ (30.7–72.9 kg/m^2^)). The following indexes were assessed in each subject: BMI, fat-free mass index (FFMI), fat mass index (FMI), tri-ponderal mass index (TMI), waist-to-height ratio (WtHR), and the body mass fat index (BMFI). Thereafter, a threshold value adjusted for age, which could identify MetS, was calculated for each index. A significant correlation was found among all indexes (*p* < 0.0001 for all). However, when the area under the curve (AUC) was compared, WtHR performed significantly better in the whole group and in the different age groups, apart from a lack of statistical difference between WtHR and BMFI in the 45–55 years age group. In conclusion, WtHR seems to be a fair index useful for identifying MetS in women with obesity. The use of thresholds appropriate for age can help further improve its accuracy, thus reinforcing the clinical evaluation for MetS screening.

## 1. Introduction

Following the pandemic rise of obesity, a significant increase has been observed in the prevalence of metabolic syndrome (MetS), this finding representing a relevant risk factor for the development of cardiovascular diseases and type 2 diabetes mellitus (T2DM). However, the risk of developing MetS is not limited to the obese population, as it can be present in a significant percentage of normal weighted individuals [1]. The frequency of MetS is generally higher in men than in women in adulthood, this pattern being reversed after the menopause when females show a progressive increase of MetS over the years [1,2]. As a consequence of this trend, elderly women, both obese and non-obese, are at higher risk and therefore need to be more closely followed longitudinally. Appropriate interventions targeted at subjects with MetS may be an important preventive care to improve health and longevity, particularly in postmenopausal women [3].

As far as the obese population is concerned, one of the main points to be clarified is which level should trigger the start of the investigations aimed to identify the presence of primary or secondary metabolic/endocrine changes. In order to address this crucial problem, on the one hand, it is mandatory to balance high cost and inconvenience to patients undergoing these investigations, while, on the other hand, there is a need to promptly detect the level of obesity triggering the development of MetS. In a recent paper, it was suggested that the body mass index (BMI), which is the most used index in clinical practice, could be considered a useful clinical threshold to help the decision [4]. However, BMI does not discriminate fat mass from fat-free mass, or the percentage of fat mass and its distribution, which in turn are considered the most important predictors of cardio-metabolic risk [5,6].

Because of these limits, other indexes have been proposed, such as the fat mass index (FMI) [7], tri-ponderal mass index (TMI) [8] (indexes of adiposity), waist circumference (WC) [9], visceral adiposity index (VAI) [10], the waist-to-height ratio (WtHR) [11] (indexes of body fat distribution), and even the fat-free mass index (FFMI) [12], which mostly reflects the nutritional status in healthy and ill subjects instead of body fat or fat distribution. The use of these surrogate indexes is mainly based on the need to adopt inexpensive methods, since the employment of instruments directly measuring fat mass and visceral adiposity, such as dual energy X-ray absorptiometry (DEXA) and magnetic resonance/computed tomography, is burdened by very high costs. However, no general agreement has been reached regarding which of these indexes performs best to date. In this context, we have recently compared these indexes plus a new one, the Body Mass Fat Index (BMFI), which also adjusts BMI for body composition and WC, for their ability to identify MetS in 1332 children and adolescents with obesity [13] and in a large group of adult subjects with the Prader-Willi syndrome (PWS) [14]. The main outcome of the two studies confirmed that BMI, which considers neither body composition nor fat distribution, performed as well as the other indexes.

The objective of this paper was to verify whether these results could also be replicated in a cohort of women with obesity over a large age range. With the background of a worsening metabolic status over the years, women with obesity are particularly at risk of developing MetS and therefore would benefit from an early diagnosis and, consequently, a prompt treatment. Therefore, early recognition of MetS or risk of MetS through easy-to-use indexes would be suitable to enable the implementation of more intensive primary prevention strategies. For this purpose, we evaluated for BMI, BMFI, FFMI, FMI, TMI, and WtHR, the age-adjusted threshold that allows to identify or exclude the presence of MetS, comparing thereafter their sensitivity and specificity, the positive and negative predicted value (PPV and NPV, respectively), and the positive and negative likelihood ratio (PLR and NLR, respectively) in order to establish which index of body composition and/or adiposity is the most reliable in identifying MetS in a large group of women with obesity.

## 2. Patients and Methods

### 2.1. Study Population

Between January 2015 and December 2018, 1528 women with obesity (aged 50.8 ± 14 years (range 18–83), BMI 43.3 ± 5.9 kg/m^2^ (30.7–72.9 kg/m^2^)), consecutively hospitalized at the Division of Metabolic Diseases, Istituto Auxologico Italiano, Piancavallo, Verbania, Italy, were studied. Inclusion criteria were BMI ≥30 kg/m^2^ and Caucasian origin. None of them suffered from secondary obesity, including genetic (e.g., monogenic and syndromic), organic (e.g., kidney or liver diseases, cerebral neoplasms), endocrine, (e.g., uncontrolled hypothyroidism, endogenous hypercortisolism, having being excluded by standard endocrine assessments (not shown)), or iatrogenic forms (e.g., chronic exposure to glucocorticoids, psychotrophic drugs). At the time of the study, 256 subjects were treated for T2DM (16.7%), while 833 subjects (54.5%) were undergoing therapy for arterial hypertension. A total of 121 patients (7.9%) were treated for dyslipidemia. A total of 54 women were previously diagnosed with T2DM, dyslipidemia, and hypertension (3.5%), 159 with T2DM and hypertension (10.4%), 49 with hypertension and dyslipidemia (3.2%), 7 with T2DM and dyslipidemia (0.4%), 36 with T2DM alone (2.3%), 571 with hypertension alone (37.4%), and 18 with dyslipidemia alone (1.2%).

Subjects were divided according to age in three subgroups: 18–44 years (*n* = 466), 45–54 years (*n* = 390), and 55+ years (*n* = 672).

The Ethical Committee of the Istituto Auxologico Italiano, Italy, approved the study protocol (Ethical Committee code: 2020_06_16_01; date of approval: 16th June 2020; acronym: ANTROINDEXOBFEM), and all patients gave their written informed consent. The study was performed in accordance with the Declaration of Helsinki and the 2005 Additional Protocol to the European Convention of Human Rights and Medicine concerning Biomedical Research.

### 2.2. Anthropometric Data

Weight and height were measured in fasting conditions after voiding. Physical examination was carried out by the same investigators, specifically trained. Standing height was determined by a Harpenden Stadiometer (Holtain Limited, Crymych, Dyfed, UK). Body weight was measured to the nearest 0.1 kg, with an electronic scale (Ro WU 150, Wunder Sa.bi., Trezzo sull’Adda, Italy), in minimal underclothes and no shoes. WC was determined in standing position midway between the lowest rib and the top of the iliac crest after gentle expiration, with a non-elastic flexible tape measure.

### 2.3. Blood Pressure Measurements and Instrumental Examinations

Diastolic and systolic blood pressure (BP) were measured to the nearest 2 mmHg in the supine position after 5 min rest, using a sphygmomanometer with an appropriately sized cuff. The average of three measurements on different days was used.

Body composition: fat mass (FM), fat mass percentage (FM%), fat-free mass (FFM), and fat-free mass percentage (FFM%) were assessed by bioelectrical impedance analysis (BIA) (Human-IM Scan; DS-Medigroup, Milan, Italy). Whole-body impedance was measured at a frequency of 50 kHz (Z_50_), following international guidelines [15]. Measurements were performed according to the method of Lukaski et al. [16], after 20-min rest in a supine position with relaxed arms and legs. The impedance index (ZI_50_) was calculated as the ratio between height (cm)^2^ and Z_50_ (Ω). In our laboratory, the within-day CV of Z_50_, as determined by 10 repeated BIA measurements of 10 obese adults, was 2% [17].

### 2.4. Laboratory Analyses

Baseline blood samples were drawn by venipuncture after a 12-h overnight fast. Standard enzymatic methods were used for measurement of glycemia, high-density lipoprotein cholesterol (HDL-C), and triglycerides (TG) (Roche Diagnostics, Mannheim, Germany).

### 2.5. Definitions

Obesity was defined in the presence of a BMI higher than 30 kg/m^2^ [18]. According to the International Diabetes Federation (IDF) criteria [19], MetS was defined in the presence of three abnormal findings out of the following five parameters: central obesity, high systolic BP and/or diastolic BP, high triglycerides, low HDL-C, and elevated fasting glucose. Central obesity was defined when WC was ≥80 cm [20]. Hypertension was defined in the presence of systolic BP values ≥130 mmHg and/or diastolic BP values ≥85 mmHg or in case of antihypertensive drugs use. Hypertriglyceridemia was defined in the presence of triglycerides values ≥150 mg/dL or in the case of a specific treatment. A low HDL-C level was defined with values <50 mg/dL. Elevated fasting glucose was defined as glycemia ≥5.6 mmol/L (or ≥100 mg/dL) or in the case of antidiabetic drugs use.

The following indexes have been calculated according to the respective formulas:

1. BMI [18]: weight (kg)/height in m^2^
2. BMFI [13]: BMI × FM (%) × WC (m)
3. FFMI [12]: fat-free mass in kg/height in m^2^
4. FMI [7]: fat mass in kg/height in m^2^
5. TMI [8]: mass in kg/height in m^3^
6. WtHR [11]: WC (cm)/height (cm)

### 2.6. Statistical Analysis

The data were first scrutinized for outliers, using a cutoff of 4.5 standard deviation score. Two patients were excluded on this basis. To explore the data, preliminary analyses were performed. A lognormality test was conducted on the logarithmic transformations of the variables used. The Kolmogorov–Smirnov test was used as a reference, with only one doubtful case for FMI, where the Kolmogorov–Smirnov test showed a *p*-value of 0.31 but a graphical analysis and the Cramer–Von–Mises test (*p* > 0.05) indicated a normal situation.

Continuous data were presented as mean ± standard deviation (SD) or with 95% confidence intervals (CIs). Mean values were tested for statistical significance using 2-tailed *t*-tests. Pearson correlation coefficients were calculated to assess the relationship between body-composition indexes. ANOVA and the Bonferroni test were used to analyze differences in clinical characteristics among the three age groups. Correlation analyses were used to assess the associations between each body-composition index and each metabolic risk factor component. Fisher’s transformation, changing r to a Z-score, was used to compare correlated correlations.

In order to calculate the growth pattern of body-composition indexes, a quantile regression was used [21] as an alternative for the LMS Method [22]. The logarithm of each body composition index was used as a response, fitted with a parametric model, which involved age^3 and age^4. The standardized residuals were retained to represent age-adjusted values.

Receiver operating characteristic (ROC) curves were then generated to obtain the values of area under the curve (AUC), with sensitivity, specificity, and 95% CI, for each age-adjusted standardized body composition index as a predictor of MetS [23]. In addition, PLR, NLR-, PPV, and NPV were examined. Adjusted ROC analysis using clinical cut points for metabolic risks was performed to identify the best predictor for each adiposity index among metabolic risk factors.

To identify the optimal cutoff, the Youden index [24] was calculated.

The significance threshold was set at *p* < 0.05. The data were analyzed using SAS Enterprise Guide 4.3 (SAS Institute Inc., Cary, NC, USA).

## 3. Results

According to the IDF criteria for MetS, all 1528 women fulfilled the criteria for abdominal obesity. High BP was detected in 1105 subjects (72.3%). Raised triglycerides values were present in 510 patients (33.4%), while 825 had reduced HDL-C levels (54.0%). Hyperglycemia was found in 505 women (33.0%). Overall, the presence of MetS was detectable in 917 patients (60.0%).

The clinical characteristics, biochemical data, and values of the different indexes of the whole population, as well as the two subgroups without (MetS−) and with MetS (MetS+), are reported in Table 1.

As expected, all parameters were significantly worse in the MetS+ group than in MetS− (*p* < 0.0001 for all but *p* < 0.01 for FMI), with the exception of FM% and FFM% (*p* = 0.88 for both). Moreover, MetS+ women were significantly older than the MetS− subjects (*p* < 0.0001).

**Figure 1:**
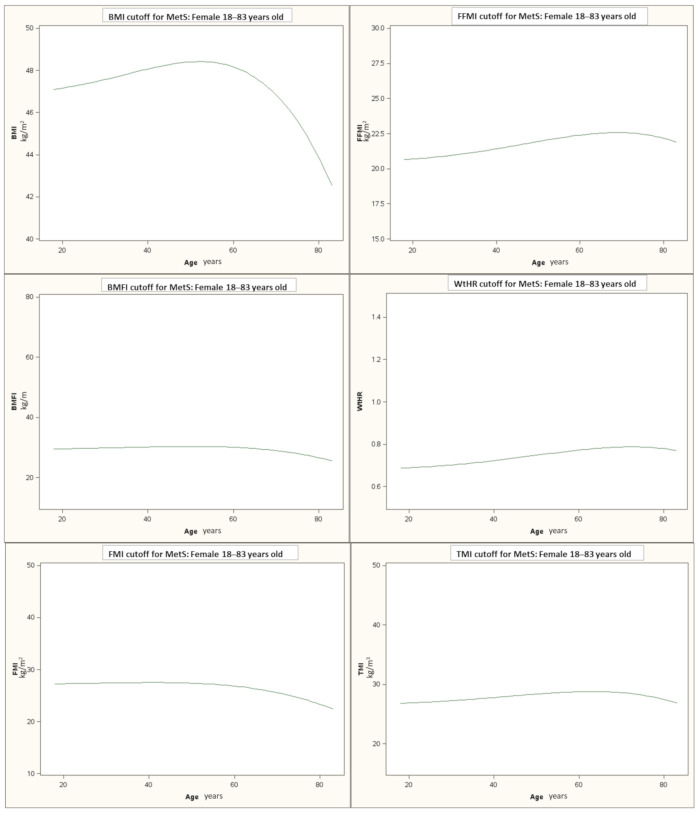
The threshold values of each index, beyond which the risk of METS is identified according to age. Abbreviations: BMI: body mass index; BMFI: body mass fat index; FMI: fat mass index; FFMI: fat-free mass index; WHtR: waist-to-height ratio; TMI: tri-ponderal mass index.

The thresholds that identify the risk of MetS for each index, according to age, are shown in Figure 1. The mean cut-off values for each index were BMI: 47.9, BMFI: 29.9, FMI: 26.9, FFMI: 21.9, WtHR: 0.75, and TMI: 28.2.

Considering the subgroup MetS+ systolic BP, glycemia and WC were significantly higher in the older age groups (Table S1, Supplementary Materials), which, on the other hand, showed higher HDL-C values.

### Correlations

Unadjusted correlations among the different indexes were all significantly correlated with each other in the whole group (*p* < 0.001 for all), in the MetS+ group (*p* < 0.001 for all), and in the MetS- group (*p* < 0.001 for all), apart from the absent correlation between FMI and FFMI (*p* = 0.08) (Table S2, Supplementary Materials). The values of correlation between the different indexes ranged from 0.98 (BMFI-FMI) and 0.41 (BMFI-FFMI) in the MetS+ and 0.96 (BMFI-FMI) and 0.12 (BMFI-FFMI) in the MetS−.

Table 2 shows the Pearson partial (age) correlation analysis between the different indexes and the components of MetS. The Fisher’s z transformation coefficients (95% CI) are reported.

Age-adjusted ROC analysis for the whole group, using clinical cut points for metabolic risks, shows that anthropometric measures are consistent predictors for hypertension and hyperglycemia (Table S3, Supplementary Materials).

In Table 3, the sensitivity, specificity, and PPV and NPV for identifying the MetS, together with the PLR and NLR for each index, are reported. WtHR was the most sensitive compared to all other indexes, while FMI and BMI showed the lowest sensitivity. The percentages of subjects that would have been diagnosed with MetS by using each of the indexes are the following: BMI: 18.2%, BMFI: 28.9%, FMI: 15.2%, FFMI: 29.1%, WtHR: 55.7%, and TMI: 37.4%.

BMI and FMI showed the highest specificity and WtHR the lowest. All indexes showed the same PPV, with only BMI superior to TMI. Considering the negative predictive value, WtHR was significantly higher than the others.

The evaluation of the PLR and NLR showed that the post-test probability was not different from the pre-test probability. The (LR+) post-test probability of MetS changed from 7% for TMI to 14% for BMI, and the (LR−) post-test probability of MetS changed from 12% for WtHR to 2% for FMI. Only with BMI and WtHR was there a small improvement in the post-test probabilities.

**Figure 2:**
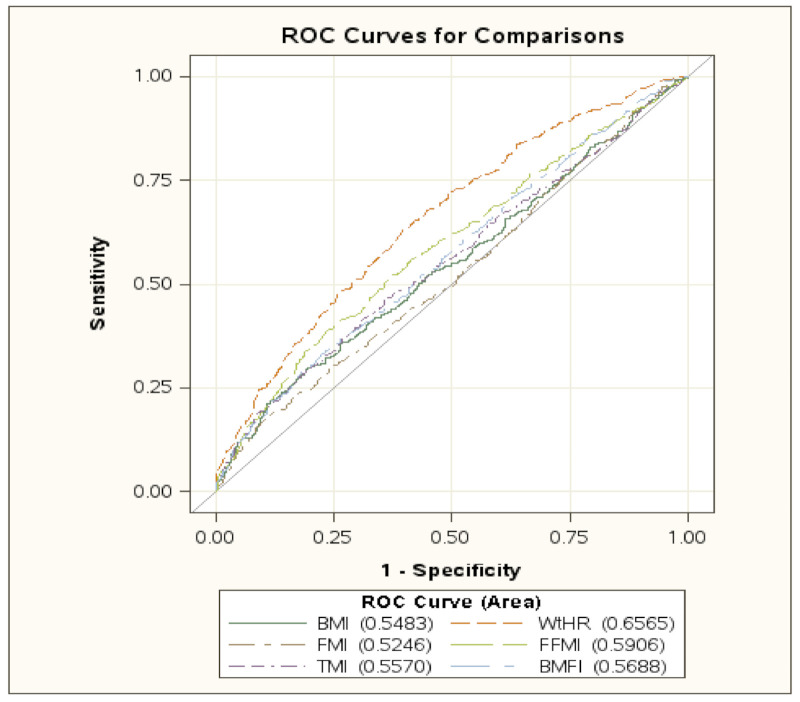
The ROC curves of the different indices in the whole population. Area under the curve for the single index is reported in brackets. Abbreviations: BMI: body mass index; WtHR: waist-to-height ratio; FMI: fat mass index; FFMI: fat-free mass index; TMI: tri-ponderal mass index; BMFI: body mass fat index.

Figure 2 (and Figure S1 in Supplementary Materials) shows the ROC curves of the different indexes in the whole population and in the three subgroups (18–44, 45–54, and 55+ years), respectively.

Table 4 shows the comparison of the different ROC curves, indicating that, in the whole group, WtHR performed significantly better than the other indexes.

Furthermore, BMI performed better than FMI, FFMI better than BMI and FMI, TMI better than FMI, and, finally, BMFI better than FMI and BMI. Analyzing the single subgroups, it is evident that, in the age range of 18–44 years, WtHR performed better than BMI, FMI, TMI, BMFI, and FFMI and BMFI performed better than FMI and BMI.

In the subgroup 45–54 years, WtHR performed better than TMI, BMI, FMI, and FFMI, while BMFI performed better than FMI, TMI, and BMI and BMI performed better than TMI.

In the subgroup 55+ years, WtHR performed better than the other indexes and BMFI performed better than FMI.

## 4. Discussion

In the present study, the accuracy of different indexes of adiposity and body composition in recognizing MetS was evaluated in a large group of women with obesity aged 18–83 years. Altogether, although a good correlation among indexes was found in the whole group, the WtHR was associated with the best performance. Furthermore, the large age span of the subjects examined allowed to demonstrate that the threshold values of different indexes, beyond which MetS was identified, varied over the years, implying that, for each age group, an appropriate cut-off should be used. This trend should be considered in the clinical praxis in order to identify the patients at risk more precisely. The age-dependent variation of the threshold values may presumably be explained by the physiological changes in body composition that usually occur in women over the years, particularly after the menopause [25], which is also characterized by a higher frequency of MetS [1]. In fact, our subgroup of women with obesity and MetS+ was older and showed more abdominal fat distribution, a condition strongly associated with the development of MetS compared to the subgroup without MetS. Furthermore, when the subgroup of MetS+ were subdivided according to age, the oldest subjects showed worsening of cardiometabolic risk factors compared to the other subgroups, with the exception of HDL-C. This last finding, fairly surprising, might be explained, at least in part, by the higher number of elderly females treated for dyslipidemia (89% of the total). In this context, although lowering cholesterol medications are known to mainly affect total and LDL cholesterol rather than HDL-C, a significant increase of HDL-C during statins therapy was recently reported [26].

In all age subgroups of women with obesity (18–44 years, 45–54 years, 55+ years), the higher performance in identifying MetS was found with the indexes, which also consider fat distribution, such as WtHR and BMFI. The analysis of correlation between the different indexes and the component of MetS confirmed that WtHR was the better predictor of the metabolic risk factors. In addition, WtHR was the more sensitive index, while BMI and FMI showed the highest specificity.

The PPV was similar for all indexes, while WtHR was slightly superior to the other indexes when NPV was considered. Altogether, according to the prevalence of MetS in the study population, the PPV was found to be higher than NPV. PLR and NLR, on the other hand, suggested that these indexes were of little help in predicting the likelihood of a true positive or negative result, with only BMI and WtHR performing slightly better.

The accuracy of the indexes was evaluated in the whole group, as well as in the different age subgroups, by comparing the ROC curves. WtHR resulted in the best one, with the exception of a lack of statistical difference between WtHR and BMFI in the 45–54 years age group.

These results were consistent with previous studies, demonstrating that WtHR was a valuable screening tool for adult cardiometabolic risk factors [27] and the most sensitive marker for predicting MetS [28,29].

In two previous papers by our group, the accuracy of the same indexes evaluated in this study was analyzed in a large group of obese children and adolescents [13] and in an adult population with PWS [14]. The conclusions of these studies were that no index performed significantly better than the others, and, therefore, BMI, which is the oldest and easiest to be calculated, should be used, even if it does not consider more sophisticate parameters, such as body composition or body fat distribution.

On the contrary, in the present study, WtHR performed slightly better than the other indexes. Several possible causes can be taken into consideration in order to explain the different results, such as the large differences in chronological age and therefore the differences in body composition and fat distribution of the present study group. As a matter of fact, associations between anthropometric indexes and regional fat depots varied between children, adolescents, and young and older adults [30]. Furthermore, PWS adults with obesity have a particular fat distribution, mostly subcutaneous, which is more advantageous from the metabolic point of view, but that does not prevent many subjects from developing MetS over the years [31]. However, this peculiar distribution of adipose tissue may be responsible for the different ability of the various anthropometric indexes to identify MetS in adults with PWS, compared to non-syndromic obesity. However, with the data in our hands, we are actually unable to give definitive answers to this discrepancy to date. In this context, we have also considered the ability of both BMI and WtHR in identifying MetS. By using our cut-offs, the combined use of BMI plus WtHR showed a sensitivity of 60% and a specificity of 52.7%, while BMI alone had a sensitivity of 22.4% and a specificity of 88.2% and WtHR alone had a sensitivity of 63% and a specificity of 60.4%. Taking these results into account, the comparable sensitivity between BMI, plus WtHR and WtHR alone, is in favour of the use of the latter. On the other hand, the high specificity of BMI alone vs BMI plus WtHR allows to exclude rather than identify MetS.

There are some weak points in our study. First of all, the main problem is that our data are derived from a cross-sectional study. It is undoubted that a longitudinal evaluation would have provided more information on the evolution over time of the ability of the different indexes taken into consideration when recognizing MetS. Another limitation is the lack of evaluation of body fat by DEXA, which would actually have been impractical and too expensive to be performed in such a large number of subjects. Moreover, the lack of analysis of our indexes in the different BMI classes (e.g., 30–40 kg/m^2^, >40–50 kg/m^2^, >50 kg/m^2^) might represent another critical issue. However, the choice to exclude this analysis was based on the markedly different numerousness of the three BMI-related subgroups (*n* = 469, *n* = 878, *n* = 181, respectively). Further additional studies with numerically homogeneous subgroups are mandatory to clarify this relevant aspect. The categorization based on age alone without actually knowing whether women were in their menopause or post-menopause might represent another limitation of the study. Although we are perfectly aware that the measurement of waist circumference may be problematic in severely obese patients, thus representing a possible bias of the present study, the examination of patients was performed by a well-trained and experienced team of a single third level center for severe obesity, thus reducing the risk of significant misinterpretations. Lastly, the present study performed on Caucasian women with obesity cannot allow to extend our conclusions to other ethnic groups.

On the other hand, the strength of this study is the huge number and large age span of the subjects, which allowed us to observe the changes of the threshold of the different indexes over the years and therefore the possibility to use them for better clinical management. In addition, the recruitment of women with obesity was made in a single center, and their examination was carried out by the same operators.

In conclusion, although most of the adiposity/body composition indexes performed adequately, the WtHR, a simple index to be calculated, performed slightly better than the others. It is of note that, similar to BMI, WtHR does not require the evaluation of body composition and therefore leads to a reduction of the costs. An important finding from this study is that, in Caucasian women with obesity, the evaluation of body composition throughout BIA does not add any additional value to simple anthropometric measures, such as BMI or WtHR, in identifying MetS. However, it should be recognized that BIA (not being as accurate as DEXA) might have influenced the present results. Finally, our results suggest that changes of the different indexes over the years should also be considered in order to ensure effective and age-appropriated rehabilitative approaches, aimed to counteract the complex comorbidities associated with obesity.

As a final remark, it must be underlined that all the indexes taken into consideration in the present study should be considered as a support for the clinicians in order to identify the presence of MetS and thus avoid unnecessary investigations. Although both WtHR and BMI represent useful, cheap, practical, and easy-to-use tools, facilitating the work of clinicians and speeding up diagnostics, it must underlined that they cannot replace the clinical judgement at all.

## Figures and Tables

**Table 1:** Descriptive statistics for all subjects, MetS−, and MetS+. Data are presented as mean ± SD. *p* is the difference between MetS− and Met+.

	All	MetS−	MetS+	*p*
Number of subjects	1528	611	917	
Age (years)	50.78 ± 14.04	46.05 ± 15.12	53.93 ± 12.31	<0.0001
SBP (mm/Hg)	128.12 ± 13.52	124.88 ± 13.1	130.28 ± 13.37	<0.0001
DBP (mm/Hg)	76.84 ± 7.57	75.91 ± 7.63	77.45 ± 7.47	<0.0001
TG (mg/dL)	132.07 ± 60.38	98.32 ± 31.17	154.55 ± 64.53	<0.0001
HDL-C (mg/dL)	50.86 ± 12.7	56.92 ± 12.17	46.81 ± 11.36	<0.0001
glycemia (mmol/L)	98.06 ± 31.72	83.59 ± 12.01	107.71 ± 36.72	<0.0001
BMI (kg/m^2^)	43.35 ± 5.93	42.55 ± 5.1	43.88 ± 6.37	<0.0001
WtHR	0.7 ± 60.08	0.74 ± 0.07	0.78 ± 0.08	<0.0001
FMI (kg/m^2^)	22.27 ± 5.16	21.82 ± 4.7	22.56 ± 5.43	<0.005
FFMI (kg/m^2^)	21.08 ± 1.82	20.73 ± 1.64	21.32 ± 1.9	<0.0001
TMI (kg/m^3^)	27.58 ± 4.08	26.98 ± 3.47	27.99 ± 4.4	<0.0001
BMFI (kg/m)	27.15 ± 8.77	25.66 ± 7.53	28.14 ± 9. 38	<0.0001
WC (cm)	119.94 ± 12.26	116.05 ± 11.34	122.54 ± 12.16	<0.0001
FM%	50.82 ± 5.35	50.8 ± 5.43	50.84 ± 5.29	0.88
FFM%	49.18 ± 5.35	49.2 ± 5.43	49.16 ± 5.29	0.88

Abbreviations: MetS− and MetS+: patients with and without metabolic syndrome; SBP: systolic blood pressure; DBP: diastolic blood pressure; TG: triglycerides; HDL-C: high-density lipoprotein cholesterol; BMI: body mass index; WHtR: waist-to-height ratio; FMI: fat mass index; FFMI: fat-free mass index; TMI: tri-ponderal mass index; BMFI: body mass fat index; WC: waist circumference; FM%: fat mass percentage; FFM%; fat-free mass percentage.

**Table 2:** Beta estimates and R^2^ values relating each anthropometric measure to cardiovascular risk outcome. All models adjust for age. The values reported for each parameter are standardized coefficient of the regression of the cardiovascular risk measures on anthropometric measures, the confidence interval of the coefficient (in brackets), and the correlation coefficient.

	SBP (mm/Hg)	DBP (mm/Hg)	HDL-C (mg/dL)	Glycemia (mg/dL)	TG (mg/dL)
BMI (kg/m^2^)	0.19 (0.14–0.23); 0.03	0.19 (0.14–0.23); 0.03	−0.08 (−0.13/−0.03); 0.01	0.09 (0.04–0.14); 0.01	−0.04 (−0.09–0.01); 0.00
WtHR	0.19 (0.14–0.24); 0.04	0.18 (0.13–0.23); 0.03	−0.09 (−0.14/−0.04); 0.01	0.1 (0.05–0.15); 0.01	−0.01 (−0.06–0.04); 0.00
FMI (kg/m^2^)	0.19 (0.14–0.24); 0.04	0.19 (0.14–0.24); 0.05	−0.05 (−0.1–0.0); 0.01	0.07 (0.02–0.12); 0.01	−0.06 (−0.11/−0.01); 0.01
FFMI (kg/m^2^)	0.08 (0.03–0.13); 0.08	0.07 (0.02–0.12); 0.07	−0.13 (−0.18/−0.08); 0.09	0.11 (0.06–0.16); 0.08	0.03 (−0.02–0.08); 0.07
TMI (kg/m^3^)	0.16 (0.11–0.21); 0.12	0.13 (0.08–0.18); 0.11	−0.15 (−0.2/−0.1); 0.12	0.16 (0.11–0.21); 0.12	0.07 (0.02–0.12); 0.1
BMFI (kg/m)	0.17 (0.12–0.22); 0.04	0.17 (0.13–0.22); 0.04	−0.07 (−0.12/−0.02); 0.01	0.1 (0.05–0.15); 0.02	−0.05 (−0.1–0.0); 0.01

Abbreviations: SBP: systolic blood pressure; DBP: diastolic blood pressure; HDL-C: high-density lipoprotein cholesterol; TG: triglycerides; BMI: body mass index; WHtR: waist-to-height ratio; FMI: fat mass index; FFMI: fat-free mass index; TMI: tri-ponderal mass index; BMFI: body mass fat index.

**Table 3:** Sensitivity, Specificity, Positive Predictive Value, Negative Predictive Value, Positive Likelihood Ratio, and Negative Likelihood Ratio for each anthropometric measure in identifying MetS.

	Sensitivity	Specificity	PPV	NPV	PLR	NLR
BMI (kg/m^2^)	22.4%	88.2%	74.0%	43.1%	1.90	0.88
BMFI (kg/m)	33.2%	77.6%	68.9%	43.6%	1.48	0.86
FMI (kg/m^2^)	18.3%	89.0%	71.5%	42.1%	1.67	0.92
FFMI (kg/m^2^)	35.1%	80.0%	72.5%	45.1%	1.76	0.81
WtHR	63.0%	60.4%	70.5%	52.1%	1.59	0.61
TMI (kg/m^3^)	41.5%	68.9%	66.7%	44.0%	1.34	0.85

Abbreviations: BMI: body mass index; BMFI: body mass fat index; FMI: fat mass index; FFMI: fat-free mass index; WHtR: waist-to-height ratio; TMI: tri-ponderal mass index; PPV: Positive Predictive Value; NPV: Negative Predictive Value; PLR: Positive Likelihood Ratio; NLR: Negative Likelihood Ratio.

**Table 4:** Adjusted ROC area, relating anthropometric measure (95% CI) in all 1528 subjects and in the different age groups.

	All Subjects	Age 18–44 Years	Age 45–54 Years	Age 55+ Years
BMI (kg/m^2^)	0.55 (0.52–0.58) °	0.51 (0.45–0.56)	0.59 (0.53–0.65) °	0.55 (0.51–0.6)
BMFI (kg/m)	0.57 (0.54–0.6) ^&^	0.54 (0.48–0.59) ^&^	0.62 (0.56–0.67) ^&^	0.57 (0.53–0.62) ^&^
FMI (kg/m^2^)	0.52 (0.5–0.55)	0.5 (0.45–0.56)	0.59 (0.53–0.64)	0.54 (0.5–0.59)
FFMI (kg/m^2^)	0.59 (0.56–0.62) ^+^	0.54 (0.48–0.59)	0.57 (0.51–0.63)	0.56 (0.51–0.61)
WtHR	0.66 (0.63–0.68) *	0.61 (0.55–0.66) *	0.64 (0.58–0.7) *	0.62 (0.58–0.67) *
TMI (kg/m^3^)	0.56 (0.53–0.59) ^	0.51 (0.45–0.56)	0.57 (0.51–0.63)	0.55 (0.5–0.6)

The values reported for each parameter are area under the curve (AUC) and 95% CI in brackets. For significance: All subjects: * *p* < 0.0001 vs. all other indexes; ° *p* < 0.0001 vs. FMI; ^+^ *p* < 0.001 vs. BMI and FMI; ^ *p* < 0.001 vs. FMI; and *p* < 0.0001 vs. FMI and *p* < 0.001 vs. BMI. Age 18–44 years: * *p* < 0.0001 vs. BMI, FMI, TMI, BMFI and *p* < 0.05 vs. FFMI; and *p* < 0.0001 vs. FMI and *p* < 0.05 vs. BMI. Age group 45–54 years: * *p* < 0.001 vs. TMI and *p* < 0.05 vs. BMI, FMI and FFMI; and *p* < 0.0001 vs. FMI, *p* < 0.01 vs. TMI and *p* < 0.05 vs. BMI; ° *p* < 0.05 vs. TMI. Age 55+ years: * *p* < 0.001 vs. BMI, FMI, TMI, *p* < 0.01 vs. BMFI and *p* < 0.05 vs. FFMI; and *p* < 0.0001 vs. FMI. Abbreviations: BMI: body mass index; BMFI: body mass fat index; FMI: fat mass index; FFMI: fat-free mass index; WtHR: waist-to-height ratio; TMI: tri-ponderal mass index.

## Data Availability

Raw data are available at https://doi.org/10.5281/zenodo.4736682 (accessed on 4 May 2021) upon a reasonable request.

## Supplementary Materials

The following are available online at https://www.mdpi.com/article/10.3390/jcm10091975/s1. Figure S1: The ROC curves of the different indices subdivided according to the age range (18–44, 45–54, 55+ years). Area under the curve for the single index is reported in brackets. Table S1: ANOVA and Bonferroni-test for all subjects MetS+, subdivided for age group. Data are presented as mean ± SD. Table S2: Pearson partial (age) correlation analysis between the different indices in all subjects, MetS− and MetS+ subjects. For significance: * *p* < 0.0001, ^&^ *p* < 0.01, ^$^ *p* = 0.056. Table S3: Area under ROC curves relating each anthropometric measure to clinical cut point after adjusting for age.
