# Supplementary material for: The Role of Different Indexes of Adiposity and Body Composition for the Identification of Metabolic Syndrome in Women with Obesity
Source: J Clin Med. 2021 May 4;10(9):1975. doi: 10.3390/jcm10091975 (PMC8125097; doi:10.3390/jcm10091975)
Supplement: Supplementary file 1 [file jcm-10-01975-s001.zip › jcm-1201322-supplementary.pdf]

Supplementary Materials

Table S1. ANOVA and Bonferroni-test for all subjects MetS +, subdivided for age group. Data are presented as mean±SD.

	18–44 Years (n = 466)	45–54 Years (n = 390)	55+ Years (n = 672)	ANOVA	18-44 vs 45-54 years	18-44 vs 55+ Years	45-54 vs 55+ Years
SBP (mm/Hg)	127.04 ± 11.75	129.04 ± 12.82	132.25 ± 13.96	<0.0001	p = 0.06;	p < 0.05	p < 0.05
DBP mm/Hg)	77.31 ± 8.08	77.39 ± 7.23	77.55 ± 7.37	0.9212	p = 0.87.	p = 0.60	p = 0.73
TG (mg/dL)	161.48 ± 71.74	157.09 ± 70.13	150.41 ± 57.75	0.1072	p = 0.36	p = 0.07	p = 0.09
HDL-C (mg/dL)	42.14 ± 9.8	46.75 ± 10.03	48.68 ± 12.11	<0.0001	p < 0.05	p < 0.05	p = 0.92
Glycemia (mg/dL)	89.97 ± 25.01	107.27 ± 32.33	114.92 ± 40.39	<0.0001	p < 0.05	p < 0.05	p < 0.05
BMI (kg/m^2)	43.9 ± 7.4	44.1 ± 6.3	43.8 ± 6.0	0.7851	p = 0.67	p = 0.80	p = 0.44

Abbreviations: SBP: systolic blood pressure; DBP: diastolic blood pressure; TG: triglycerides; HDL-C: high-density lipoprotein cholesterol; BMI: body mass index.

Table S2. Pearson partial (age) correlation analysis between the different indices in all subjects, MetS- and MetS+ subjects.

	All Subjects, N = 1528						MetS– Subjects, N = 611						MetS+ Subjects, N = 917					
	**BMI**	**WtHR**	**FMI**	**FFMI**	**TMI**	**BMFI**	**BMI**	**WtHR**	**FMI**	**FFMI**	**TMI**	**BMFI**	**BMI**	**WtHR**	**FMI**	**FFMI**	**TMI**	**BMFI**
BMI (kg/m^2)	1	0.72*	0.96*	0.55*	0.96*	0.94*	1	0.63*	0.95*	0.39*	0.94*	0.92*	1	0.77*	0.96*	0.62*	0.96*	0.95*
WtHR	0.72*	1	0.65*	0.51*	0.76*	0.78*	0.63*	1	0.55*	0.38*	0.69*	0.72*	0.77*	1	0.72*	0.55*	0.80*	0.81*
FMI (kg/m^2)	0.96*	0.65*	1	0.27*	0.87*	0.97*	0.95*	0.55*	1	0.08$	0.83*	0.96*	0.96*	0.72*	1	0.38*	0.90*	0.98*
FFMI (kg/m^2)	0.55*	0.51*	0.27*	1	0.64*	0.33*	0.39*	0.38*	0.08$	1	0.54*	0.12&	0.62*	0.55*	0.38*	1	0.68*	0.41*
TMI (kg/m^3)	0.96*	0.76*	0.87*	0.64*	1	0.86*	0.94*	0.69*	0.83*	0.54*	1	0.80*	0.96*	0.80*	0.90*	0.68*	1	0.88*
BMFI (kg/m)	0.94*	0.78*	0.97*	0.33*	0.86*	1	0.92*	0.72*	0.96*	0.12&	0.80*	1	0.95*	0.81*	0.98*	0.41*	0.88*	1

For significance: *p < 0.0001, &p < 0.01, $$p$ = 0.056. Abbreviations: BMI: body mass index; WHtR: waist-to-height ratio; FMI: fat mass index; FFMI: fat-free mass index; TMI: tri-ponderal mass index; BMFI: body mass fat index.

Table S3. Area under ROC curves relating each anthropometric measure to clinical cutpoint, after adjusting for age.

	**Hypertension (SBP > 130 mmHg and/or DBP > 85 mmHg)**	**Hypertriglyceridemia (> 150 mg/dL)**	**HypoHDL-C (< 50 mg/dL)**	**Glycemia (> 100 mg/dL)**
BMI (kg/m²)	0.80	0.57	0.57	0.70
BMFI (kg/m)	0.80	0.57	0.58	0.71
FMI (kg/m²)	0.80	0.58	0.57	0.70
FFMI (kg/m²)	0.79	0.57	0.58	0.70
WtHR	0.80	0.59	0.60	0.72
TMI (kg/m³)	0.80	0.57	0.57	0.70

Abbreviations: BMI: body mass index; BMFI: body mass fat index; FMI: fat mass index; FFMI: fat-free mass index; WHtR: waist-to-height ratio; TMI: tri-ponderal mass index.

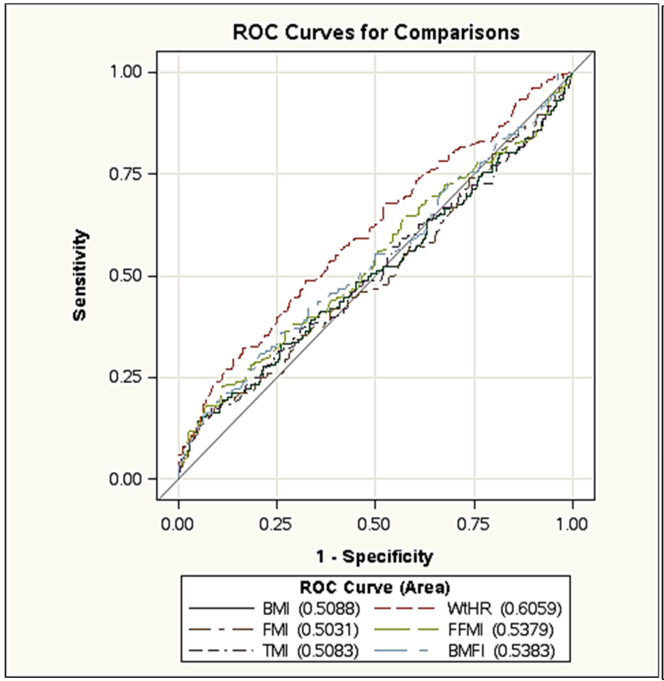

Age: 18–44

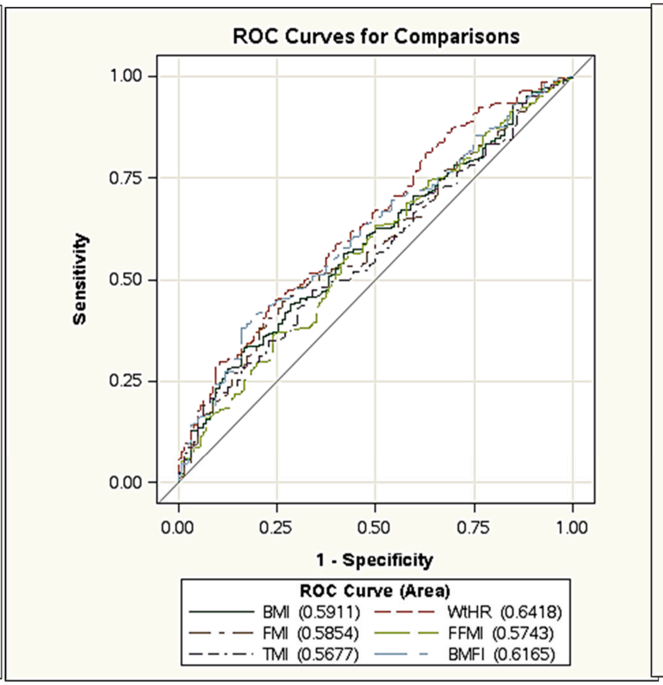

Age 44–54

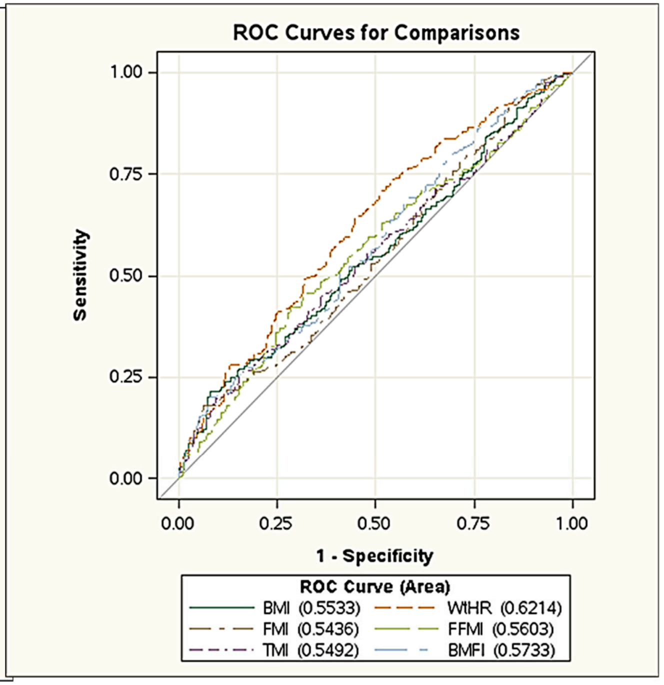

Age 55+

Figure S1. The ROC curves of the different indices subdivided according to the age range (18-44, 45-54, 55+ years). Area under the curve for the single index is reported in brackets.
Abbreviations: BMI: body mass index; WHtR: waist-to-height ratio; FMI: fat mass index; FFMI: fat-free mass index; TMI: tri-ponderal mass index; BMFI: body mass fat index.
